# Two-By-One model of cytoplasmic incompatibility: Synthetic recapitulation by transgenic expression of *cifA* and *cifB* in *Drosophila*

**DOI:** 10.1371/journal.pgen.1008221

**Published:** 2019-06-26

**Authors:** J. Dylan Shropshire, Seth R. Bordenstein

**Affiliations:** 1 Department of Biological Sciences, Vanderbilt University, Nashville, TN, United States of America; 2 Vanderbilt Microbiome Initiative, Vanderbilt University, Nashville, TN, United States of America; 3 Department of Pathology, Microbiology, and Immunology, Vanderbilt University, Nashville, TN, United States of America; 4 Vanderbilt Institute for Infection, Immunology and Inflammation, Vanderbilt University Medical Center, Nashville, TN, United States of America; The University of Melbourne, AUSTRALIA

## Abstract

*Wolbachia* are maternally inherited bacteria that infect arthropod species worldwide and are deployed in vector control to curb arboviral spread using cytoplasmic incompatibility (CI). CI kills embryos when an infected male mates with an uninfected female, but the lethality is rescued if the female and her embryos are likewise infected. Two phage WO genes, *cifA*_*wMel*_ and *cifB*_*wMel*_ from the *w*Mel *Wolbachia* deployed in vector control, transgenically recapitulate variably penetrant CI, and one of the same genes, *cifA*_*wMel*_, rescues wild type CI. The proposed Two-by-One genetic model predicts that CI and rescue can be recapitulated by transgenic expression alone and that dual *cifA*_*wMel*_ *and cifB*_*wMel*_ expression can recapitulate strong CI. Here, we use hatch rate and gene expression analyses in transgenic *Drosophila melanogaster* to demonstrate that CI and rescue can be synthetically recapitulated in full, and strong, transgenic CI comparable to wild type CI is achievable. These data explicitly validate the Two-by-One model in *w*Mel-infected *D*. *melanogaster*, establish a robust system for transgenic studies of CI in a model system, and represent the first case of completely engineering male and female animal reproduction to depend upon bacteriophage gene products.

## Author summary

Releases of *Wolbachia*-infected mosquitos are underway worldwide because *Wolbachia* block replication of Zika and Dengue viruses and spread themselves maternally through arthropod populations via cytoplasmic incompatibility (CI). The CI drive system depends on a *Wolbachia*-induced sperm modification that results in embryonic lethality when an infected male mates with an uninfected female, but this lethality is rescued when the female and her embryos are likewise infected. We recently reported that the phage WO genes, *cifA* and *cifB*, cause the sperm modification and *cifA* rescues the embryonic lethality caused by the *w*Mel *Wolbachia* strain deployed in vector control. These reports motivated proposal of the Two-by-One model of CI whereby two genes cause lethality and one gene rescues it. Here we provide unequivocal support for the model in the *Wolbachia* strain used in vector control via synthetic methods that recapitulate CI and rescue in the absence of a *Wolbachia* infections. Our results reveal the set of phage WO genes responsible for this powerful genetic drive system, act as a proof-of-concept that these genes alone can induce gene drive like crossing patterns, and establish methodologies and hypotheses for future studies of CI in *Drosophila*. We discuss the implications of the Two-by-One model towards functional mechanisms of CI, the emergence of incompatibility between *Wolbachia* strains, vector control applications, and CI gene nomenclature.

## Introduction

*Wolbachia* are the most widespread endosymbiotic bacteria on the planet and are estimated to infect half of all arthropod species [1,2] and half of the Onchocercidae family of filarial nematodes [3]. They specialize in infecting the cells of reproductive tissues, are primarily inherited maternally from ova to offspring, and often act in arthropods as reproductive parasites that enhance their maternal transmission by distorting host sex ratios and reproduction [4,5]. The most common type of reproductive parasitism is cytoplasmic incompatibility (CI), which manifests as a sperm modification in infected males that causes embryonic lethality or haploidization in matings with uninfected females upon fertilization [6–8]. This embryonic lethality is rescued if the female is infected with the same *Wolbachia* strain. As such, CI selfishly drives CI-inducing *Wolbachia* into host populations [9–13], and the incompatibilities between host populations cause reproductive isolation between recently diverged or incipient species [14–18].

In the last decade, *Wolbachia* and CI have garnered significant interest for their utility in combatting vector borne diseases worldwide. Two strategies are currently deployed: population suppression and population replacement. The population suppression strategy markedly crashes vector population sizes through the release of only infected males that induce CI upon mating with wild uninfected females [19–22]. In contrast, the population replacement strategy converts uninfected to infected populations through the release of both infected males and females that aid the spread *Wolbachia* via CI and rescue [23,24]. Replacing a vector competent, uninfected population with infected individuals can notably reduce the spread of arthropod borne diseases such as Zika and dengue [25,26] because *Wolbachia* appear to inhibit various stages of viral replication within arthropods based on diverse manipulations of the host cellular environment [27–33]. The combination of *Wolbachia*’s abilities to suppress arthropod populations, drive into host populations, and block the spread of viral pathogens have established *Wolbachia* in the vanguard of vector control efforts to curb arboviral transmission [22–25,34–36].

An unbiased, multi-omic analysis of CI-inducing and CI-incapable *Wolbachia* strains revealed two adjacent genes, *cifA* and *cifB*, in the eukaryotic association module of prophage WO [37] that strictly associate with CI induction [38]. Fragments of the CifA protein were found in the fertilized spermathecae of *w*Pip infected *Culex pipiens* mosquitoes [39], and these genes are frequently missing or degraded in diverse CI-incapable strains [40,41]. Dual transgenic expression of *cifA* and *cifB* from either of the CI-inducing strains *w*Mel or *w*Pip in uninfected male flies causes a decrease in embryonic hatching corresponding to an increase in CI-associated cytological abnormalities including chromatin bridging and regional mitotic failures [38,42]. Single transgenic expression of either *cifA*_*wMel*_ or *cifB*_*wMel*_ in an uninfected male was insufficient to recapitulate CI, but single transgenic expression of either gene in an infected male enhances *w*Mel-induced CI in a dose-dependent manner [38]. Importantly, dual transgenic CI induced by *cifA*_*wMel*_ and *cifB*_*wMel*_ expressing males was rescued when they were mated with *w*Mel-infected females [38]. Moreover, transgenic expression of *cifA*_*wMel*_ alone in uninfected females rescues embryonic lethality and nullifies cytological defects associated with wild type CI caused by a *w*Mel infection [43].

As such, we recently proposed the Two-by-One genetic model of CI wherein dual expression of *cifA*_*wMel*_ and *cifB*_*wMel*_ causes CI when expressed in males and expression of *cifA*_*wMel*_ rescues CI when expressed in females [43]. However, confirmation of the model’s central prediction requires the complete synthetic replication of CI-induced lethality and rescue in the absence of any *Wolbachia* infections since it remains possible that other *Wolbachia* or phage WO genes besides *cifA* and *cifB* contribute to wild type CI and rescue by *w*Mel *Wolbachia*. Moreover, CI induced by dual *cifA*_*wMel*_ and *cifB*_*wMel*_ expression previously yielded variable offspring lethality with a median survival of 26.5% of embryos relative to survival of 0.0% of embryos from CI induced by a wild type infection under controlled conditions [38]. The inability to recapitulate strong wild type CI suggests other CI genes are required, other environmental factors need to be controlled, or the transgenic system requires optimization.

**Fig 1:**
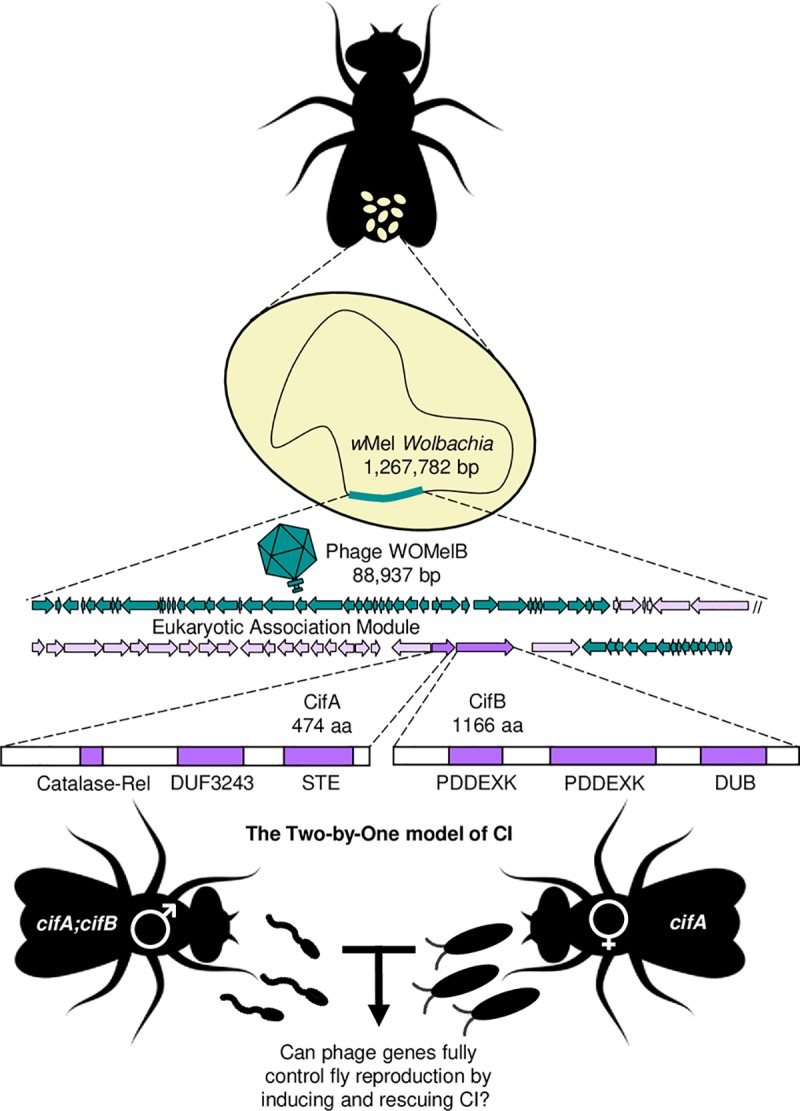
Two-by-One model of CI is governed by *cifA* and *cifB* genes in the eukaryotic association module of prophage WO in *Wolbachia*. The Two-by-One model of CI predicts that *D*. *melanogaster* males and females can be engineered to recapitulate both CI and rescue phenotypes in the absence of *Wolbachia*, thus depending completely on phage genes for successful reproduction. Schematics are not to scale. Insect, sperm, and embryo art were obtained and modified using vecteezy.com. Phage gene schematics modified from [38]. CifA and CifB protein annotation from [40]. Purple indicates eukaryotic association module genes as indicated by [37].

Here, we utilize transgenic expression, hatch rates, and gene expression assays in *Drosophila melanogaster* to test if an optimized expression system can generate strong transgenic CI and whether bacteriophage genes *cifA*_*wMel*_ and *cifB*_*wMel*_ can fully control fly reproduction by inducing and rescuing CI in the complete absence of *Wolbachia* (Fig 1). We further assess if both *cif*_*wMel*_ genes are required for CI induction in the optimized system and whether *cifA*_*wMel*_ in females can rescue transgenic CI. Results provide strong evidence for the Two-by-One model in *w*Mel-infected *D*. *melanogaster*, offer context for conceptualizing CI mechanisms and the evolution of bidirectional incompatibilities between different *Wolbachia* strains, raise points for CI gene nomenclature, and motivate further research in developing these genes into a tool that combats vector borne diseases. To the best of our knowledge, they also represent the first case of completely engineering animal sexual reproduction to depend upon bacteriophage gene products.

## Results

### Optimizing transgenic CI

Dual transgenic expression of *cifA*_*wMel*_ and *cifB*_*wMel*_ was previously reported to induce highly variable and incomplete CI relative to CI caused by an age-controlled *w*Mel infection [38], indicating either the presence of other genes necessary for strong CI, environmental factors uncontrolled in the study, or inefficiency of the transgenic system. Here, we test the latter hypothesis by dually expressing *cifA*_*wMel*_ and *cifB*_*wMel*_ in uninfected *D*. *melanogaster* males under two distinct GAL4 driver lines that express in reproductive tissues: *nos*-GAL4-*tubulin* and *nos*-GAL4:VP16 [44]. Both driver lines contain a *nos* promoter region, but differ in that *nos*-GAL4-*tubulin* produces a transcription factor with both the DNA binding and transcriptional activating region of the GAL4 protein, and *nos*-GAL4:VP16 produces a fusion protein of the GAL4 DNA binding domain and the virion protein 16 (VP16) activating region [45,46]. The GAL4:VP16 transcription factor is a particularly potent transcriptional activator because of its binding efficiency to transcription factors [47,48]. Additionally, the *nos*-GAL4-*tubulin* driver has a *tubulin* 3’ UTR, and *nos*-GAL4:VP16 has a *nos* 3’ UTR that may contribute to differences in localization within cells or between tissues [44–46]. As such, we predict that differences in the expression level or profile of these two driver lines will lead to differences in the penetrance of transgenic CI.

**Fig 2:**
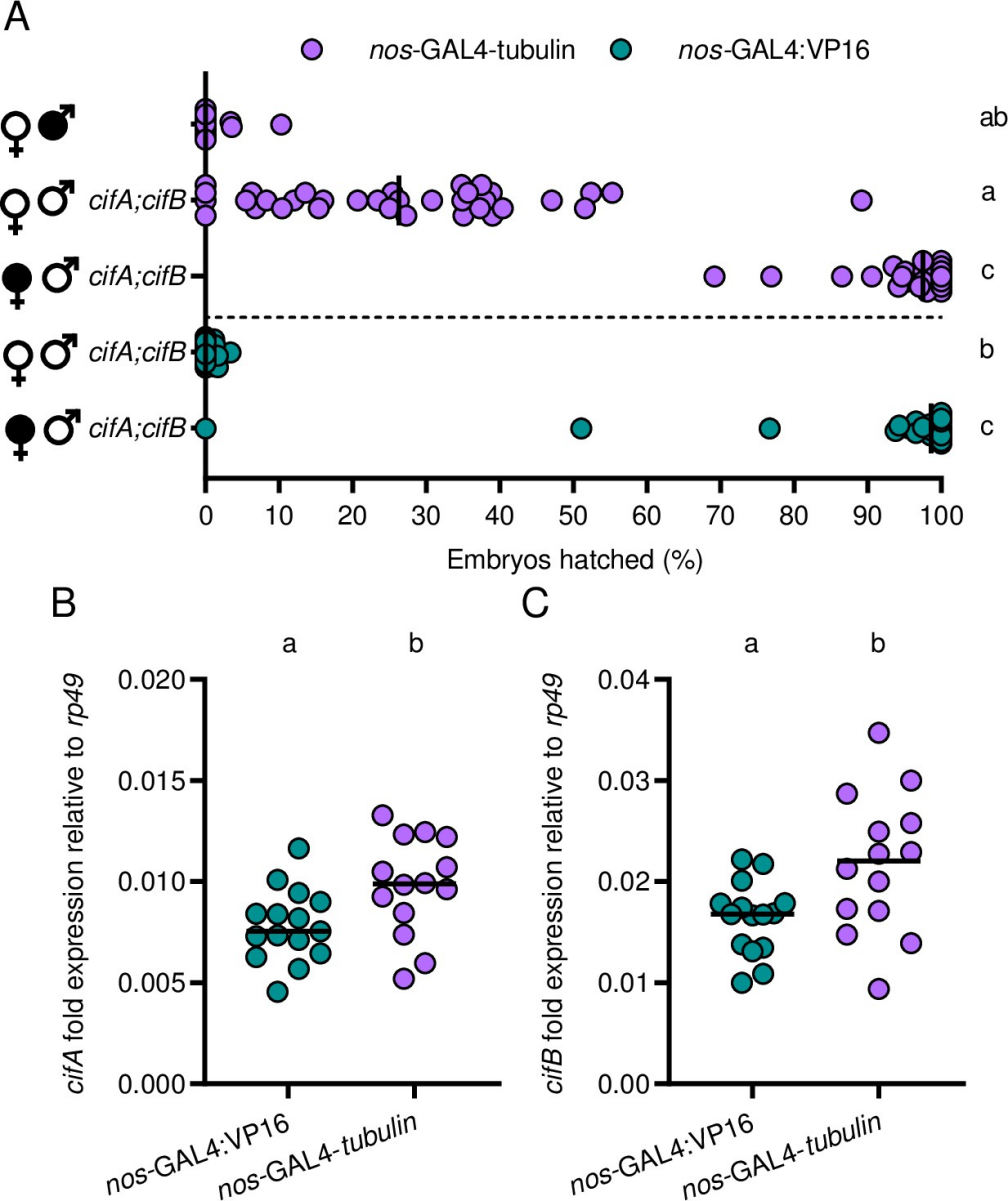
*cifA*_*wMel*_ and *cifB*_*wMel*_ induce strong CI when transgenically expressed in males under the *nos*-GAL4:VP16 driver. (A) Two different driver lines, *nos*-GAL4-*tubulin* (purple; top) and *nos*-GAL4:VP16 (green; bottom) were tested for their ability to induce CI when transgenically expressed in uninfected, male *Drosophila*. Filled sex symbols represent infection with *w*Mel *Wolbachia*, and gene names to the right of a symbol represent expression of those genes in the male line. Vertical bars represent medians. Letters to the right indicate significant differences with an α = 0.05 calculated by a Kruskal-Wallis analysis followed by Dunn’s multiple comparison test. (B,C) To test if *nos*-GAL4-*tubulin* and *nos*-GAL4:VP16 generate different levels of gene expression, (B) *cifA*_*wMel*_ and (C) *cifB*_*wMel*_ fold expression difference relative to the *Drosophila* housekeeping gene *rp49* in male abdomens under the two drivers was measured using qPCR. Males tested for gene expression were the same used in the hatch rate experiment in A. Letters above indicate significant differences with an α = 0.05 calculated by a Mann-Whitney U test.

Since CI manifests as embryonic lethality, we measure hatching of *D*. *melanogaster* embryos into larvae to quantify the strength of CI. We confirm previous findings [38] that dual transgenic expression of *cifA*_*wMel*_ and *cifB*_*wMel*_ under *nos*-GAL4-*tubulin* in uninfected males yields low but variable embryonic hatching in crosses with uninfected females (Mdn = 26.3%, IQR = 10.4–38.1%) that can be rescued in crosses with *w*Mel-infected females (Mdn = 97.5%; IQR = 94.2–100%) (Fig 2A). However, dual *cifA*_*wMel*_ and *cifB*_*wMel*_ expression under *nos*-GAL4:VP16 in uninfected males yields significantly reduced embryonic hatching relative to *nos*-GAL4-*tubulin* (p = 0.0002) with less variability (Mdn = 0%; IQR = 0.0–0.75%) and can be comparably rescued (Mdn = 98.65%; IQR = 95.93–100%; p > 0.99) (Fig 2A). Together, these results support that dual *cifA*_*wMel*_ and *cifB*_*wMel*_ expression under *nos*-GAL4:VP16 induces the strongest CI and that the transgenic system, not the absence of necessary CI factors, contributed to the prior inability to recapitulate strong wild type CI.

Next, we tested the hypothesis that differences in the penetrance of transgenic CI between the two drivers are due to differences in the strength of expression. To assess this, we used qPCR to measure the gene expression of *cifA*_*wMel*_ and *cifB*_*wMel*_ under the two drivers relative to a *Drosophila* housekeeping gene (*rp49*) in male abdomens (Fig 2B and 2C). Fold differences in RNA transcripts of *cifA*_*wMel*_ relative to *rp49* reveal *nos*-GAL4-*tubulin* (Mdn = 0.0098; IQR = 0.0082–0.122) drives significantly stronger and more variable *cifA*_*wMel*_ expression relative to *nos*-GAL4:VP16 (Mdn = 0.0075; IQR = 0.0064–0.0090) (p = 0.016, MWU, Fig 2B). The same is true for *cifB*_*wMel*_ expression where *nos*-GAL4-*tubulin* (Mdn = 0.022; IQR = 0.0165–0.0265) drives significantly stronger *cifB*_*wMel*_ expression than *nos*-GAL4:VP16 (Mdn = 0.0168; IQR = 0.0135–0.0179) (p = 0.02, MWU, Fig 2C). Moreover, while *cifA*_wMel_ and *cifB*_*wMel*_ expression significantly correlate with each other under both *nos*-GAL4-*tubulin* (R^2^ = 0.85; p <0.0001) and nos-GAL4:VP16 (R^2^ = 0.75; p <0.0001; S1A Fig), neither *cifA*_wMel_ (R^2^ = 0.02; p = 0.62; S1B Fig) nor *cifB*_*wMel*_ (R^2^ = 0.04; p = 0.48; S1C Fig) expression levels under the *nos*-GAL4-*tubulin* driver correlate with the strength of CI measured via hatch rates. Notably, *cifB*_*wMel*_ is consistently more highly expressed than *cifA*_*wMel*_ within the same line (S1A Fig). We predict that expression differences are due to either differences in transgenic insertion sites or more rapid degradation of *cifA*_*wMel*_ relative to *cifB*_*wMel*_. Taken together, these results suggest that an increase in CI penetrance in these crosses is not positively associated with higher transgene transcript abundance from different drivers.

### Optimizing transgenic rescue

**Fig 3:**
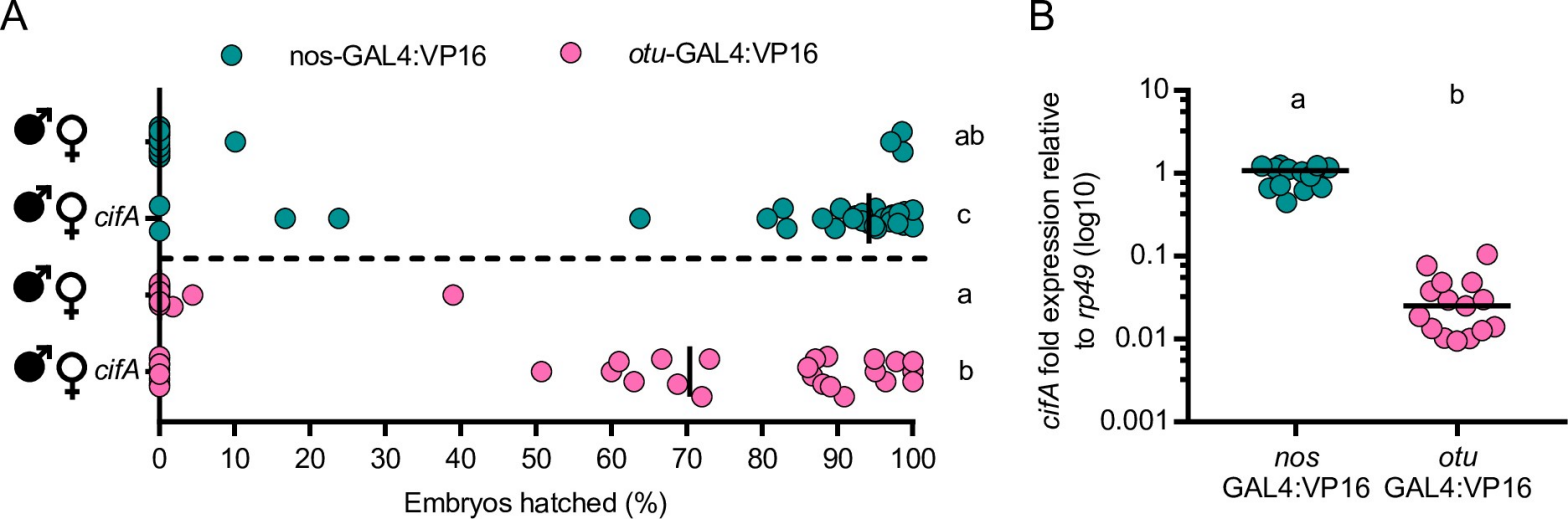
*cifA*_*wMel*_ can induce strong rescue when expressed in uninfected females under the *nos*-GAL4:VP16 driver. (A) Two different driver lines, *nos*-GAL4:VP16 (green; top) and *otu*-GAL4:VP16 (pink; bottom), were tested for their ability to rescue *w*Mel induced CI. Filled sex symbols represent infection with *w*Mel *Wolbachia*, and gene names to the right of a symbol represent expression of those genes in the corresponding sex of that cross. Vertical bars represent medians. Letters to the right indicate significant differences with an α = 0.05 calculated by a Kruskal-Wallis analysis followed by Dunn’s multiple comparison test. (B) To test if nos-GAL4-tubulin and nos-GAL4:VP16 generate different levels of RNA expression, *cifA*_*wMel*_ fold expression difference relative to the *Drosophila* housekeeping gene *rp49* in male abdomens under the two drivers was measured using qPCR. Females tested for gene expression were the same used in the hatch rate experiment in A. Letters above indicate significant differences with an α = 0.05 calculated by a Mann-Whitney U test.

*cifA*_*wMel*_ expression under the maternal triple driver (MTD) in uninfected females can rescue CI induced by a wild type infection [43]. MTD is comprised of three drivers in the same line: *nos*-GAL4-*tubulin*, *nos*-GAL4:VP16, and *otu*-GAL4:VP16 [44]. We previously reported that *cifA*_*wMel*_ expression under the *nos*-GAL4-*tubulin* driver alone is rescue-incapable [43]. Here, we test if *cifA*_*wMel*_ expression under either of the other components of the MTD driver independently recapitulate rescue of *w*Mel CI. Hatch rate experiments indicate that CI is strong and expectedly not rescued when an infected male mates with a non-transgenic female whose genotype is otherwise *nos*-GAL4:VP16 (Mdn = 0.0%; IQR = 0.0–0.0%) or *otu*-GAL4:VP16 (Mdn = 0.0%; IQR = 0.0–0.0%) (Fig 3A). Transgenic expression of *cifA*_*wMel*_ in uninfected females under either of the two drivers rescues CI induced by *w*Mel. However, rescue is significantly weaker under *cifA*_*wMel*_ expression with the *otu*-GAL4:VP16 driver (Mdn = 70.4%; IQR = 0.0–90.45%) as compared to the *nos*-GAL4:VP16 driver (Mdn = 94.2%; IQR = 83.3–97.1%; p = 0.0491) which produced strong transgenic rescue (Fig 3A). Gene expression analysis of *cifA*_wMel_ relative to *rp49* in the abdomens of uninfected females reveals that *nos*-GAL4:VP16 expresses *cifA*_wMel_ significantly higher (Mdn = 1.08; p < 0.0001) than *otu*-GAL4:VP16 (Mdn = 0.03) (Fig 3B), suggesting that high expression in females may underpin the ability to rescue. Alternatively, *nos*-GAL4:VP16 and *otu*-GAL4:VP16 are known to express GAL4 at different times in oogenesis, with the former in all egg chambers and the latter in late stage egg chambers [44].

### The Two-by-One model of CI

**Fig 4:**
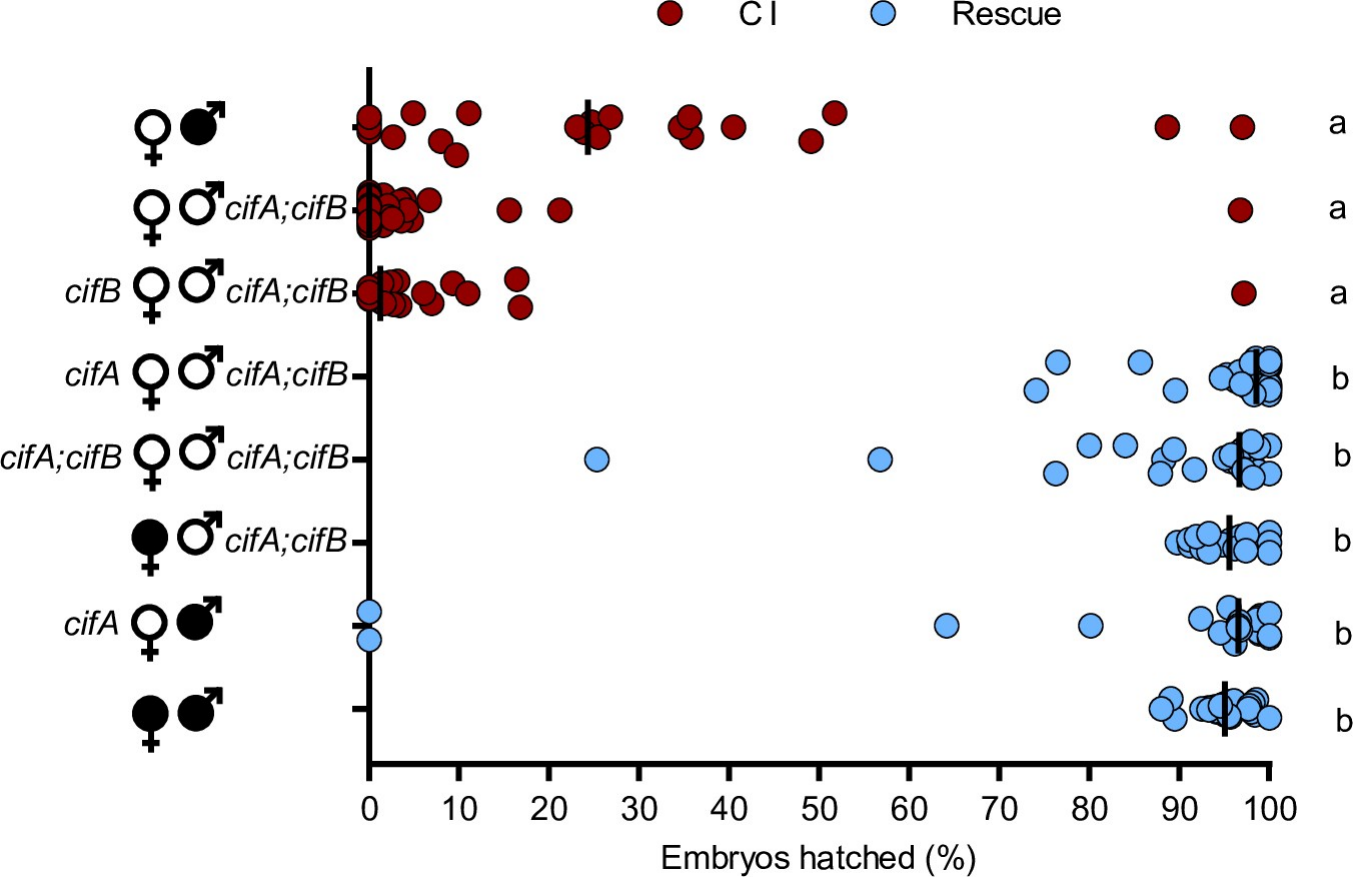
CI and rescue can be synthetically recapitulated under transgenic expression in the absence of *Wolbachia*. Single *cifA*_*wMel*_ and dual *cifA*_*wMel*_ *and cifB*_*wMel*_ expression under *nos*-GAL4:VP16 in uninfected females (open circles) were tested for their ability to rescue transgenic CI under the same driver in uninfected males. Filled sex symbols represent infection with *w*Mel *Wolbachia*, and gene names beside a symbol represent expression of those genes in the corresponding sex of that cross. Vertical bars represent medians. Letters to the right indicate significant differences with an α = 0.05 calculated by a Kruskal-Wallis analysis followed by Dunn’s multiple comparison test.

With the transgenic expression system optimized for both transgenic CI and rescue, we then tested the hypothesis that the Two-by-One model can be synthetically recapitulated by dual *cifA*_*wMel*_ and *cifB*_*wMel*_ expression in uninfected males to cause CI and single *cifA*_*wMel*_ expression in uninfected females to rescue that transgenic CI. Indeed, dual *cifA*_*wMel*_ and *cifB*_*wMel*_ expression in uninfected males causes hatch rates comparable to wild type CI (Mdn = 0.0%; IQR = 0.0%-2.55; p > 0.99) (Fig 4). Transgenic CI cannot be rescued by single *cifB*_*wMel*_ expression in uninfected females (Mdn = 1.25%; IQR = 0.0–3.35%). Transgenic CI can be rescued by single *cifA*_*wMel*_ expression (Mdn = 98.6%; IQR = 97.35–100%; p = 0.41) or dual *cifA*_*wMel*_ and *cifB*_*wMel*_ expression (Mdn = 96.7%; IQR = 88.3–98.2%; p > 0.99) to levels comparable to rescue from a wild type infection (Mdn = 95.6%; IQR = 92.5–97.4%). In addition, *cifA*_*wMel*_ rescues a wild type infection at comparable levels to wild type rescue (Mdn = 96.6%; IQR = 93.5–98.85%; p > 0.99). These data provide strong evidence for the Two-by-One model in *w*Mel-infected *D*. *melanogaster*, namely that CI induced by transgenic dual *cifA*_*wMel*_ and *cifB*_*wMel*_ expression is sufficient to induce strong CI, and that *cifA*_*wMel*_ alone is sufficient to rescue it.

**Fig 5:**
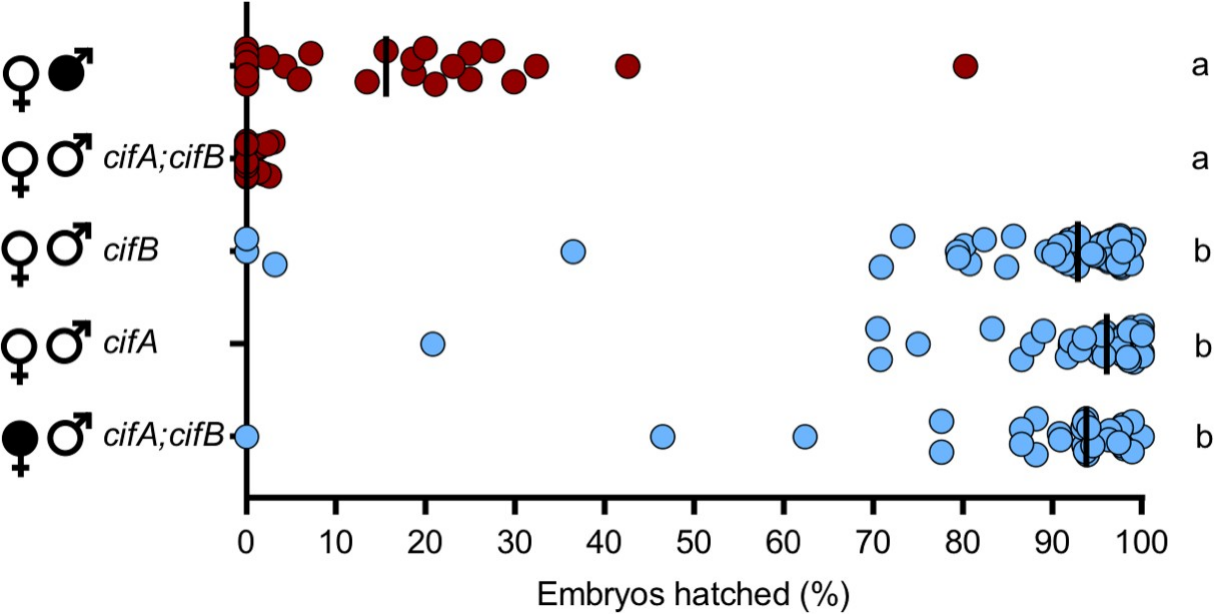
Neither *cifA*_*wMel*_ or *cifB*_*wMel*_ alone can induce CI when expressed under *nos*-GAL4:VP16. *cifA*_*wMel*_ and *cifB*_*wMel*_ were tested for their ability to induce CI individually under nos-GAL4:VP16 expression in uninfected males (open circles). Filled sex symbols represent infection with *w*Mel *Wolbachia* and gene names to the right of a symbol represent expression of those genes in the corresponding sex of that cross. Vertical bars represent medians. Letters to the right indicate significant differences with an α = 0.05 calculated by a Kruskal-Wallis analysis followed by Dunn’s multiple comparison test.

Next we reevaluated if single *cifA*_*wMel*_ or *cifB*_*wMel*_ expression under the more potent *nos*-GAL4:VP16 driver in uninfected males can recapitulate CI. Hatch rates indicate that dual *cifA*_*wMel*_ and *cifB*_*wMel*_ expression induces strong transgenic CI (Mdn = 0.0%; IQR = 0.0–1.15%) that can be rescued by a wild type infection (Mdn = 93.8%; IQR = 88.2–97.4%), whereas single expression of *cifA*_*wMel*_ (Mdn = 96.1%; IQR = 97.78–98.55%; p < 0.0001) or *cifB*_*wMel*_ (Mdn = 92.85%; IQR = 84.28–96.4%; p < 0.0001) failed once again to produce embryonic hatching comparable to expressing both genes together (Fig 5). In one replicate experiment, we note a statistically insignificant (p = 0.182) decrease in hatching under *cifB*_*wMel*_ expression relative to wild type rescue cross (S1 Data file). Thus, both *cifA*_wMel_ and *cifB*_*wMel*_ are required for strong CI. Together, these and earlier results validate the Two-by-One model of CI in *w*Mel whereby *cifA*_*wMel*_ and *cifB*_*wMel*_ expression are required and sufficient for strong CI, while *cifA*_*wMel*_ expression is sufficient to rescue it.

## Discussion

CI is the most common form of *Wolbachia*-induced reproductive parasitism and is currently at the forefront of vector control efforts to curb transmission of dengue, Zika, and other arthropod-borne human pathogens [22–25,34,35]. Two prophage WO genes from *w*Mel *Wolbachia* cause CI (*cifA*_*wMel*_ and *cifB*_*wMel*_) and one rescues wild type CI (*cifA*_*wMel*_) [38,43], supporting the proposal of a Two-by-One model for the genetic basis of CI [43]. However, dual transgenic expression of *cifA*_*wMel*_ and *cifB*_*wMel*_ recapitulates only weak and highly variable CI as compared to CI induced by a wild type infection [38]. In addition, the Two-by-One model predicts that both CI and rescue can be synthetically recapitulated by dual *cifA*_*wMel*_ and *cifB*_*wMel*_ expression in uninfected males and *cifA*_*wMel*_ expression in uninfected females. Here we optimized the transgenic system for CI and rescue by these genes, further validated the necessity of expressing both *cifA*_*wMel*_ and *cifB*_*wMel*_ for CI, and synthetically recapitulated the Two-by-One model for CI with transgenics in the absence of *Wolbachia*.

CI induced by *w*Mel *Wolbachia* can be highly variable and correlates with numerous factors including *Wolbachia* density [49], *cifA*_*wMel*_ and *cifB*_*wMel*_ expression levels [38], host age [50–52], mating rate [50], rearing density [53], development time [53], and host genetic factors [52,54–56]. Some of these factors, such as age, are known to also correlate with the level of *cif*_*wMel*_ gene expression [38]. As such, we hypothesized that prior reports of weakened transgenic CI could be explained by low levels of transgenic *cifA*_*wMel*_ and *cifB*_*wMel*_ expression in male testes [38].

Indeed, strong CI with a median of 0% embryonic hatching was induced when both *cifA*_*wMel*_ and *cifB*_*wMel*_ were expressed under the *nos*-GAL4:VP16 driver. However, contrary to our expectations, *nos*-GAL4:VP16 generates significantly weaker *cifA*_*wMel*_ and *cifB*_*wMel*_ expression than the *nos*-GAL4-*tubulin* driver previously used to recapitulate weak CI [38]. Thus, the expression data conflict with previous reports in mammalian cells wherein the GAL4:VP16 fusion protein is a more potent transcriptional activator than GAL4 [48]. Other differences between the two driver constructs may explain phenotypic differences, including the presence of different 3’ UTRs that may contribute to differences in transcript localization [44]. While it remains possible, though unlikely, that other *Wolbachia* or phage WO genes may contribute to CI, the induction of near complete embryonic lethality confirms that *cifA*_*wMel*_ and *cifB*_*wMel*_ are sufficient to transgenically induce strong CI and do not require other *Wolbachia* or phage WO genes to do so. Moreover, comparative multi-omics demonstrated that *cifA* and *cifB* are the only two genes strictly associated with CI capability [38].

We previously recapitulated transgenic rescue of *w*Mel-induced CI by expression of *cifA*_*wMel*_ under the Maternal Triple Driver (MTD) [43], which is comprised of three independent drivers [44]. Expression of *cifA*_*wMel*_ using one of the MTD drivers in flies was previously shown to be rescue-incapable [43]; the other drivers had not been evaluated. Here, we tested the hypothesis that expression of *cifA*_*wMel*_ using either of the two remaining drivers is sufficient to rescue CI, and we found that *cifA*_*wMel*_ expression under both driver lines recapitulates rescue, but at different strengths. Indeed, rescue is strongest when *cifA*_*wMel*_ transgene expression is highest. These data are consistent with reports that *cifA*_*wMel*_ is a highly expressed gene in transcriptomes of *w*Mel-infected females [57] and the hypothesis that rescue capability is largely determined by the strength of *cifA*_*wMel*_ expression in ovaries [43]. These results combined with those for transgenic expression of CI now establish a robust set of methods for future studies of transgene-induced CI and rescue in the *D*. *melanogaster* model.

**Fig 6:**
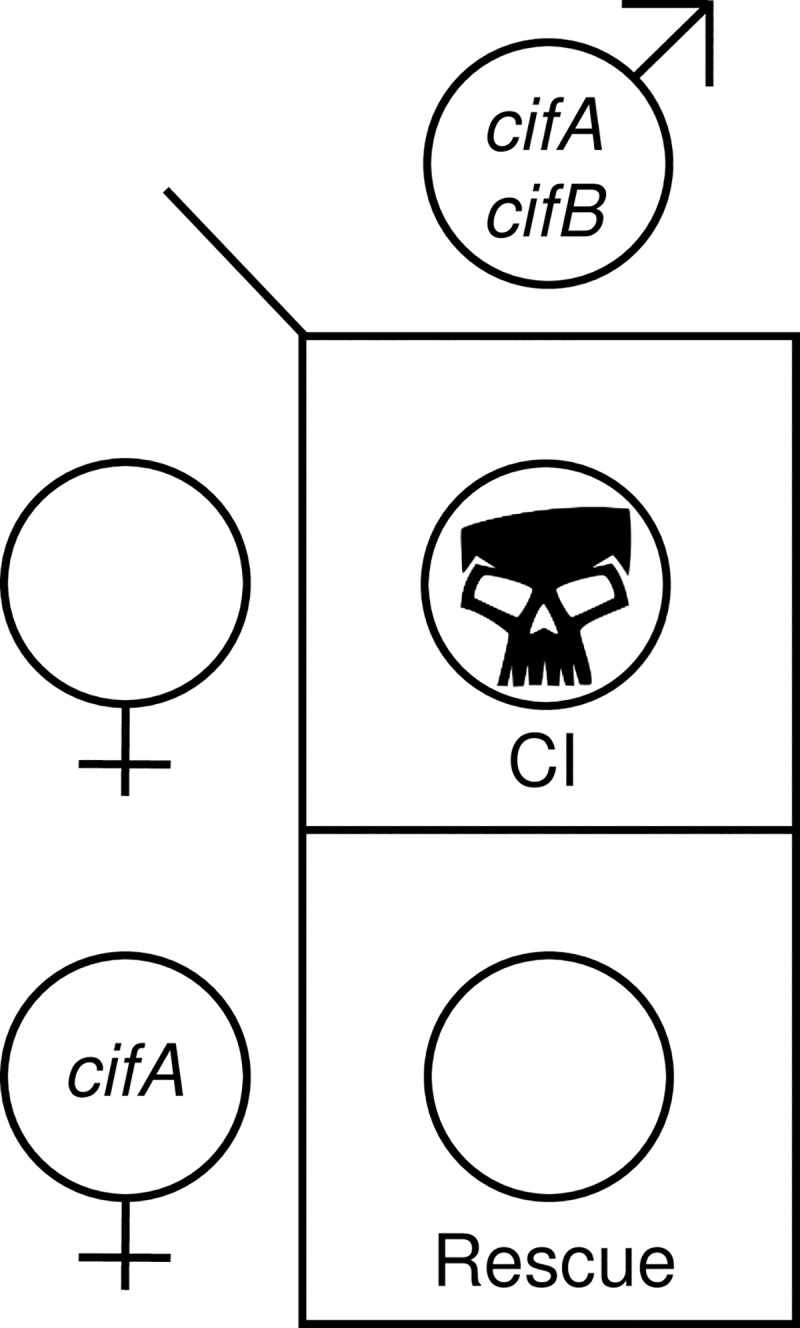
The Two-by-One model of CI. The Two-by-One genetic model of CI explains that *cifA* and *cifB* dual expression in uninfected males is necessary for embryonic lethality (CI; skull) when crossed to uninfected and non-expressing females. However, females expressing *cifA* can rescue CI in their offspring (rescue; open circle). Skull art is from vecteezy.com.

The central prediction of the Two-by-One model is that transgenic CI can be synthetically rescued in the absence of *Wolbachia* through dual *cifA* and *cifB* expression in uninfected males and *cifA* expression in uninfected females. Here, we explicitly validate the model that two genes are required in males to cause CI, and one in females is required to rescue it using *w*Mel *cif* gene variants. However, to confirm that the optimized expression system does not influence the ability of *cifA*_*wMel*_ or *cifB*_*wMel*_ alone to induce CI, we singly expressed them with the improved driver and found that embryonic hatching does not statistically differ from compatible crosses. Coupled with prior data in *w*Mel [38,43], these results strongly support the Two-by-One genetic model whereby dual *cifA*_*wMel*_ and *cifB*_*wMel*_ expression is required in the testes to cause a sperm modification that can then be rescued by *cifA*_*wMel*_ expression in the ovaries (Fig 6A).

While the genetic basis of unidirectional CI appears resolved, it remains unclear how *cifA*_*wMel*_ and *cifB*_*wMel*_ functionally operate to generate these phenotypes. Numerous mechanistic models have been proposed over the last two decades [58–64]. We can broadly summarize these models into either host-modification (HM) [59] or toxin-antidote (TA) [58] models. HM models suggest that CI-inducing factors modify host products in such a way that would be lethal unless they are later reversed by rescue factors [59–64]. Conversely, TA models state that the CI-inducing factor is toxic to the developing embryo unless it is crucially bound to a cognate antidote provided by the female [42,58,59]. There are numerous lines of evidence in support of both sets of hypotheses and while the Two-by-One genetic model does not explicitly support or favor one set of models over the other, it can be used to generate hypotheses related to the mechanism of CI.

HM models [59] predict that CI factors directly interact with host products in the testes, modify them, and are displaced. These modifications travel with the sperm, in the absence of *Wolbachia* and Cif products, and would induce the canonical cytological embryonic defects including delayed paternal nuclear envelope breakdown, slowed Cdk1 activation, a failure of maternal histones to deposit onto the paternal genome, stalled or failed replication of the paternal DNA, a failure of paternal chromosomes to segregate, and later stage regional mitotic failures [7,38,60,61,64–67], or they are reversed by female-derived rescue factors. Leading HM models are the Mistiming [60,61] and Goalkeeper [63] models that leverage findings that male pronuclei are delayed in the first mitosis during embryonic development in CI crosses [61,65,67]. Since the first mitosis is initiated when the female pronucleus has developed, the delay of the male pronuclei leads to cytological defects [60]. It is thus proposed that rescue occurs through resynchronization of the first mitosis by comparably delaying the female pronucleus [60,61]. The Goalkeeper model expands the mistiming model to propose that the strength of the delay is what drives incompatibility between different *Wolbachia* strains [63]. There are numerous hypotheses to explain the role of the Cif products in these kinds of models. One such hypothesis would be that CifA is responsible for pronuclear delay, thus capable of delaying both the male and female pronuclei, but it requires CifB to properly interact with testis-associated targets. This hypothesis may predict that CifB acts to either protect CifA from ubiquitin tagging and degradation, localize it to a host target, or bind CifA to elicit a conformational change required for interacting with male-specific targets. Alternatively, CI-affected embryos express defective paternal histone deposition, protamine development, delayed nuclear breakdown, and delays in replication machinery [7,60,61,64–67]. Any of these factors could be explained by modifications occurring from HM-type interactions between Cif and host products.

TA models [58] contrast to HM models and require that the CI toxin transfers with or in the sperm and directly binds to a female-derived antidote in the embryo. If the antidote is absent, the CI toxin would induce cytological embryonic defects [7,38,60,61,64–67]. There is mixed evidence in support of this model. First, mass spectometry and SDS-PAGE analyses in *Culex pipiens* reveal that CifA_wPip_ peptides are present in female spermatheca after mating, suggesting CifA_wPip_ is transferred with or in the sperm [39]. CifB_wPip_ was not detected in these analyses, curiously suggesting that the CifB toxin was not transferred [39]. These results are inconsistent with the TA model, but the lack of transferred CifB may occur because *cifB* gene expression is up to nine-fold lower than that of *cifA* [57], and the concentration may have been too low to be observed via these methods. Second, CifA and CifB bind in vitro [42]. However, it remains unclear if CifA-CifB binding enables rescue since this binding has no impact on known enzymatic activities of CifB [42]. While the Two-by-One model does not explicitly support or reject the TA model, it does further inform it. Most intriguing is to understand how CifA acts as a contributor to CI when expressed in testes and as a rescue factor when expressed in ovaries. One hypothesis is that CifA and CifB bind to form a toxin complex that is later directly inhibited by female derived CifA [43,59]. The difference in function between these two environments could be explained by post-translational modification and/or differential localization of CifA in testes and embryos [43,59]. Alternatively, CifB may be the primary toxin, but is incapable of inducing CI unless a CifA antidote is present in both the testes and the ovaries [58]. This hypothesis predicts that male-derived CifA rapidly degrades, leaving CifB with or in the sperm. On its own, CifB would induce lethal cytological embryonic defects [60–62,64] unless provided with a fresh supply of CifA from the embryo.

It has been suggested that divergence in CI and rescue factors causes the incipient evolution of reciprocal incompatibility, or bidirectional CI, between different *Wolbachia* strains [38,43,68,69]. Here, we review a non-exhaustive set of hypotheses that we previously proposed to explain the emergence of bidirectional CI and are consistent with the Two-by-One model [43]. First, the simplest explanation for CifA’s role in both CI and rescue is that it has similar functional effects in both testes/sperm and ovaries/embryos. Thus, instead of requiring a separate mutation for CI and another for rescue [69], bidirectional CI may emerge from a single CifA mutation that causes incompatibility against the ancestral strain while maintaining self-compatibility. Second, CifA in testes and ovaries may also have different functions, localizations, or posttranslational modifications that contribute to CI and rescue. If this occurs, or if CifB is also an incompatibility factor, the evolution of bidirectional CI may require two or more mutations, and the strain may pass through an intermediate phenotype wherein it becomes unidirectionally incompatible with the ancestral variant or loses the capability to induce either CI or rescue before becoming bidirectionally incompatible with the ancestral variant. In fact, some *Wolbachia* strains are incapable of inducing CI but capable of rescuing CI induced by other strains [70], and some can induce CI but cannot be rescued [71]. Furthermore, sequence variation in both *cifA* and *cifB* from *Wolbachia* strains in *Drosophila* [38] and in small regions among strains of *w*Pip *Wolbachia* [68] have been correlated to incompatibility, suggesting that variation in both genes influence incompatibility.

Additionally, it remains possible that significant divergence in *cifA*, *cifB*, or both may be necessary to generate new phenotypes. Indeed, comparative genomic analyses reveal high levels of amino acid divergence in CifA and CifB that correlates with incompatibility between strains [38,40]. Moreover, some *Wolbachia* strains harbor numerous phage WO variants, each with their own, often divergent, *cif* genes, and the presence of multiple variants likewise correlates with incompatibility [38,40,68]. Thus, horizontal transfer of phage WO [37,72–76] can in theory rapidly introduce new compatibility relationships, and duplication of phage WO regions, or specifically *cif* genes, in the same *Wolbachia* genome may relax the selective pressure on the *cif* genes and enable their divergence. Determining which of the aforementioned models best explains the evolution of incompatibilities between *Wolbachia* strains will be assisted by additional sequencing studies to identify incompatible strains with closely related *cif* variants.

The genetic bases of numerous gene drives have been elucidated in plants [77], fungi [78–81], and nematodes [82,83]. Some gene drives have also been artificially replicated with transgenic constructs [84–86]. However, to our knowledge, the synthetic replication of the Two-by-One model of CI represents the first instance that a gene drive has been constructed by engineering eukaryotic reproduction to depend on phage proteins. Additionally, vector control programs using *Wolbachia* rely on their ability to suppress pathogens such as Zika and dengue viruses, reduce the size of vector populations, and spread *Wolbachia* into a host population via CI and rescue. However, there are limitations to these approaches. Most critically, not all pathogens are inhibited by *Wolbachia* infection and some are enhanced, such as West Nile Virus in *Culex tarsalis* infected with *w*AlbB *Wolbachia* [87]. Additionally, it requires substantial effort to establish a *Wolbachia* transinfection in a target non-native species [88] that could be obviated in genetically tractable vectors utilizing transgenic gene drives.

The complete synthetic replication of CI and rescue via the Two-by-One model represents a step towards transgenically using the *cif* genes in vector control efforts. The separation of CI mechanism from *Wolbachia* infection could theoretically expand CI’s utility to spread ‘payload’ genes that reduce the vectoral capacity of their hosts [89] into a vector population by, for instance, expressing the CI genes and the payload gene polycistronically under the same promoter in the vector’s nuclear or mitochondrial genomes. Moreover, these synthetic constructs have potential to increase the efficiency of *Wolbachia*-induced CI if they are transformed directly into *Wolbachia* genomes. For these efforts to be successful, considerable work is necessary to (i) generate a constitutively expressing *cif* gene drive that does not require GAL4 to operate, (ii) understand the spread dynamics of transgenic CI, (iii) characterize the impact of *cif* transgenic expression on insect fitness relative to wild vectors, (iv) generate and test effective payload genes in combination with *cif* drive, (v) explore and optimize the efficacy of *cif* drive in vector competent hosts such as mosquitoes, (vi) assess the impact of host factors on *cif* drive across age and development, (vii) compare the efficacy of a *cif* gene drive to other comparable technologies (CRISPR, homing drive, Medea, etc), in addition to numerous other lines of study. For example, while a substantial body of literature exists to describe the spread dynamics of CI [10,12,13,36,90,91], none yet describe how the Two-by-One model would translate into nuclear or mitochondrial spread dynamics in the absence of *Wolbachia*. As such, this study represents an early proof of concept that these genes alone are capable of biasing offspring survival in favor of flies expressing these genes under strictly controlled conditions, and should motivate additional study towards its application in vector control.

The generality of the Two-by-One model remains to be tested because it may be specific to certain strains of *Wolbachia* and/or phage haplotypes. For instance, transgenic expression of *cifB*_*wPip*_ from *C*. *pipiens* in yeast yields temperature sensitive lethality that can be rescued by dual-expression of *cifA*_*wPip*_ and *cifB*_*wPip*_ [42]. Moreover, attempts to generate a *cifB*_*wPip*_ transgenic line failed, possibly due to generalized toxicity from leaky expression [42]. Therefore, *cifB*_*wPip*_ alone could in theory cause CI. However, this model has not been explicitly tested, it has not been explained how *cifA*_*wPip*_ and *cifB*_*wPip*_ dual-expression induces CI in transgenic *Drosophila* but prevents CI in yeast, and transgenic *w*Pip CI has not been rescued in an insect. As such, it remains possible that *cifB*_*wPip*_ lethality could be explained by artefactual toxicity of overexpression or toxic expression in a heterologous system. Thus, confirmation of an alternative model for CI in *w*Pip is precluded by lack of evidence that *cifB*_*wPip*_ alone can induce rescuable lethality in an insect. Since *cifB*_*wPip*_ transgenic UAS constructs have not been generated due to toxicity from leaky expression, alternative PhiC31 landing sites or expression systems (i.e., the Q System) could prove valuable in addressing these questions.

Finally, these results further validate the importance of *cifA*_*wMel*_ as an essential component of CI and underscore a community need to unify the nomenclature of the CI genes. When the CI genes were first reported, they were described as both CI factors (*cif*) and as CI deubiquitilases (*cid*), both of which are actively utilized in the literature. The *cif* nomenclature was proposed as a cautious naming strategy agnostic to the varied biochemical functions to be discovered, whereas the *cid* nomenclature was proposed based on the finding that the B protein is in part an *in vitro* deubiquitilase that, when ablated, inhibits CI-like induction [38,42]. A recent nomenclature proposal suggested that the *cif* gene family name be used as an umbrella label to describe all CI-associated factors whereas *cidA* and *cidB* would be used to describe the specific genes [58]. However, we do not agree with this nomenclature revision despite the appeal of combining the two nomenclatures. CifA protein is not a putative deubiquitilase [40], does not influence deubiquitilase activity of CifB [42], functions independently to rescue CI [43] and, as emphasized by the work in this study, is necessary for CI induction and rescue. The competing nomenclature presumes that it is appropriate to name the A protein *cid* because it could be expressed in an operon with the B protein. However, the evidence for the operon status of the genes is weak, and more work is needed to describe the regulatory control of these genes before they can be categorized as an operon [59]. Moreover, distant homologs that cluster into distinct phylogenetic groups are proposed to be named CI nucleases (*cin*) [42] yet the merger of these two groups into one name lacks phylogenetic rationality as the two lineages are as markedly divergent from each other as they are from *cid* [59]. In addition, none of these distant homologs have been functionally characterized as CI genes [38,40]. As such, it is more appropriate to call these genes “*cif*-like” to reflect their homology and unknown phenotypes. Thus, the holistic and conservative *cif* nomenclature with Types (e.g., I-IV) used to delineate phylogenetic clades is appropriately warranted in utilizing and unifying CI gene naming.

In conclusion, the results presented here support that both *cifA*_*wMel*_ and *cifB*_*wMel*_ phage genes are necessary and sufficient to induce strong CI. In addition, *cifA*_*wMel*_ is the only gene necessary for rescue of either transgenic or wild type *w*Mel CI. These results confirm the Two-by-One model of CI in *w*Mel *Wolbachia* and phage WO with implications for the mechanism of CI and for the diversity of incompatibility between strains, and they provide additional context for understanding CI currently deployed in vector control efforts. The synthetic replication of CI in the absence of *Wolbachia* marks an early step in developing CI as a tool for genetic and mechanistic studies in *D*. *melanogaster* and for vector control efforts that may drive payload genes into vector competent populations.

## Materials and methods

### Fly rearing and strains

*D*. *melanogaster* stocks *y*^1^w* (BDSC 1495), *nos-*GAL4-*tubulin* (BDSC 4442), *nos*-GAL4:VP16 (BDSC 4937), *otu*-GAL4:VP16 (BDSC 58424), and UAS transgenic lines homozygous for *cifA*, *cifB*, and *cifA;B* [38] were maintained at 12:12 light:dark at 25^o^ C and 70% relative humidity (RH) on 50 ml of a standard media. *cifA* insertion was performed with *y*1 M{vas-int.Dm}ZH-2A w*; P{CaryP}attP40 and *cifB* insertion was performed with *y*1 *w*67c23; P{CaryP}attP2, as previously described [38]. UAS transgenic lines and *nos*-GAL4:VP16 were uninfected whereas *nos-*GAL4-*tubulin* and *otu*-GAL4:VP16 lines were infected with *w*Mel *Wolbachia*. Uninfected versions of infected lines were produced through tetracycline treatment as previously described [38]. WolbF and WolbR3 primers were regularly used to confirm infection status [38]. Stocks for virgin collections were stored at 18^o^ C overnight to slow eclosion rate, and virgin flies were kept at room temperature.

### Hatch rate assays

To test for CI, hatch rate assays were used as previously described [38,43]. Briefly, GAL4 adult females were aged 9–11 days post eclosion and mated with UAS males. Age controlled GAL4-UAS males and females were paired in 8 oz bottles affixed with a grape-juice agar plate smeared with yeast affixed to the opening with tape. 0–48 hour old males were used since CI strength rapidly declines with male age [50,52]. The flies and bottles were stored at 25^o^ C for 24 h at which time the plates were replaced with freshly smeared plates and again stored for 24 h. Plates were then removed and the number of embryos on each plate were counted and stored at 25^o^ C. After 30 h the remaining unhatched embryos were counted. The percent of embryos hatched into larvae was calculated by dividing the number of hatched embryos by the initial embryo count and multiplying by 100.

### Expression analyses

To assay transgenic RNA expression levels under the various gene drive systems, transgene expressing flies from hatch rates were immediately collected and frozen at -80°C for downstream application as previously described [43]. In brief, abdomens were dissected, RNA was extracted using the Direct-zol RNA MiniPrep Kit (Zymo), the DNA-free kit (Ambion, Life Technologies) was then used to remove DNA contamination, and cDNA was generated with SuperScript VILO (Invitrogen). Quantitative PCR was performed on a Bio-Rad CFX-96 Real-Time System in duplicate using iTaq Universal SYBR Green Supermix (Bio-Rad) using the cifA_opt and rp49 forward and reverse primers as previously described [43]. Samples with a standard deviation >0.3 between duplicates were excluded from analysis. Fold expression of *cifA* relative to *rp49* was determined with 2^−ΔΔCt^. Each expression study was conducted once.

### Statistical analyses

All statistical analyses were conducted in GraphPad Prism (Prism 8). Hatch rate statistical comparisons were made using Kruskal-Wallis followed by a Dunn’s multiple comparison test. A Mann-Whitney-U was used for statistical comparison of RNA fold expression. A linear regression was used to assess correlations between hatch rate and expression. All p-values are reported in S1 Table.

## Supporting information

S1 Table P-values associated with all statistical comparisons made in main and supporting information figures. (XLSX)

S1 Fig Fold expression of transgenic *cifA*_*wMel*_ correlates with *cifB*_*wMel*_ in males relative to the *Drosophila* housekeeping gene *rp49* but neither correlate with hatch rate under the *nos*-GAL4-tubulin driver. (A) A linear regression of *cifA*_*wMel* and_ *cifB*_*wMel*_ expression reveals a positive correlation for both *nos*-GAL4-*tubulin* and *nos*-GAL4VP16. (B,C) A linear regression of (B) *cifA*_*wMel*_ and (C) *cifB*_*wMel*_ expression and embryonic hatching reveals no correlation for *nos*-*GAL4*-*tubulin*. Removal of data points corresponding to 0% embryonic hatching did not change the significance of the correlation. The nos-GAL4:VP16 driver was not included in analysis A or B since the majority of data points corresponded with 0% hatching. This analysis uses hatch rate samples from the experiment in Fig 2A and expression data from Fig 2B and Fig 2C.
(TIF)

S1 Data File All data associated with figures and replicate experiments. (XLSX)
