# Supplementary figures and images for: Two-By-One model of cytoplasmic incompatibility: Synthetic recapitulation by transgenic expression of cifA and cifB in Drosophila
Source: PLoS Genet. 2019 Jun 26;15(6):e1008221. doi: 10.1371/journal.pgen.1008221 (PMC6594578; doi:10.1371/journal.pgen.1008221)

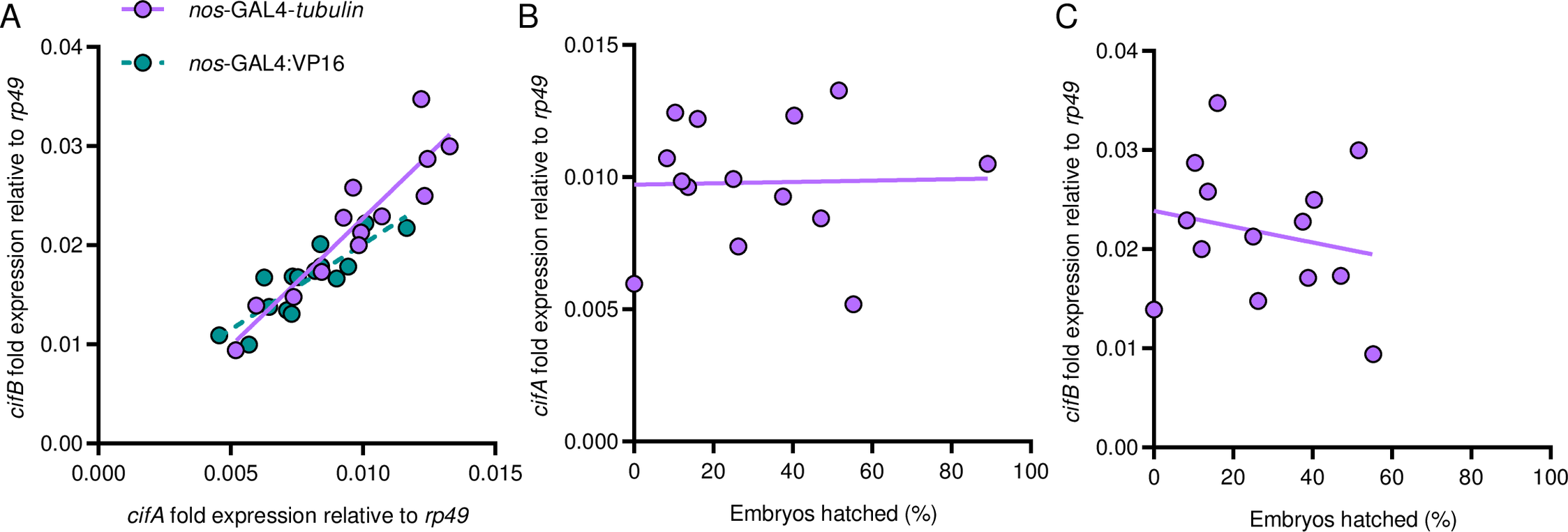

Supplement: S1 Fig — (A) A linear regression of cifAwMel and cifBwMel expression reveals a positive correlation for both nos-GAL4-tubulin and nos-GAL4VP16. (B,C) A linear regression of (B) cifAwMel and (C) cifBwMel expression and embryonic hatching reveals no correlation for nos-GAL4-tubulin. Removal of data points corresponding to 0% embryonic hatching did not change the significance of the correlation. The nos-GAL4:VP16 driver was not included in analysis A or B since the majority of data points corresponded with 0% hatching. This analysis uses hatch rate samples from the experiment in Fig 2A and expression data from Fig 2B and Fig 2C. (TIF) [file pgen.1008221.s002.tif]
